# Neuroprotective Effects of Aldehyde-Reducing Composition in an LPS-Induced Neuroinflammation Model of Parkinson’s Disease

**DOI:** 10.3390/molecules28247988

**Published:** 2023-12-07

**Authors:** Sora Kang, Youngjin Noh, Seung Jun Oh, Hye Ji Yoon, Suyeol Im, Hung Taeck Kwon, Youngmi Kim Pak

**Affiliations:** 1 Department of Neuroscience, Graduate School, Kyung Hee University, Seoul 02447, Republic of Korea; ksr8947@khu.ac.kr (S.K.); yhj2271@khu.ac.kr (H.J.Y.); 2 Picoentech Co., Ltd., Seongnam-si 13201, Gyeong gi-do, Republic of Korea; yjnoh0614@picoentech.com (Y.N.); thomas@picoentech.com (H.T.K.); 3 Department of Biomedical Sciences, Graduate School, Kyung Hee University, Seoul 02447, Republic of Korea; ohsungjun124@khu.ac.kr (S.J.O.); suryeol@khu.ac.kr (S.I.); 4 Department of Physiology, College of Medicine, Kyung Hee University, Seoul 02447, Republic of Korea

**Keywords:** ALDH, oxidative stress, neuroinflammation, Parkinson’s disease

## Abstract

Parkinson’s disease (PD) is a complex neurodegenerative disease in which neuroinflammation and oxidative stress interact to contribute to pathogenesis. This study investigates the in vivo neuroprotective effects of a patented yeast extract lysate in a lipopolysaccharide (LPS)-induced neuroinflammation model. The yeast extract lysate, named aldehyde-reducing composition (ARC), exhibited potent antioxidant and anti-aldehyde activities in vitro. Oral administration of ARC at 10 or 20 units/kg/day for 3 days prior to intraperitoneal injection of LPS (10 mg/kg) effectively preserved dopaminergic neurons in the substantia nigra (SN) and striatum by preventing LPS-induced cell death. ARC also normalized the activation of microglia and astrocytes in the SN, providing further evidence for its neuroprotective properties. In the liver, ARC downregulated the LPS-induced increase in inflammatory cytokines and reversed the LPS-induced decrease in antioxidant-related genes. These findings indicate that ARC exerts potent antioxidant, anti-aldehyde, and anti-inflammatory effects in vivo, suggesting its potential as a disease-modifying agent for the prevention and treatment of neuroinflammation-related diseases, including Parkinson’s disease.

## 1. Introduction

Parkinson’s disease (PD) is a neurodegenerative disorder characterized by the selective loss of dopaminergic neurons in a specific region of the brain in the pars compacta of the substantia nigra (SN) and striatum (ST) [1,2]. This results in a deficiency of dopamine, a crucial neurotransmitter regulating movement and other cognitive functions [3]. Dopaminergic neurons are particularly susceptible to oxidative damage due to the natural dopamine metabolism, generating reactive oxygen species (ROS). This oxidative stress affects various macromolecules, including α-synuclein, contributing to cellular and mitochondrial dysfunction [4]. The interplay of oxidative stress and neuroinflammation creates a harmful feedback loop, further damaging neurons and playing a pivotal role in PD pathogenesis [5]. However, the precise mechanisms that cause PD are not yet fully understood and further research is needed to fully elucidate its causes.

Neuroinflammation is a major pathological feature of PD and has therefore been suggested to play a key role in the development and progression of the disease [6,7]. Neuroinflammation in PD is characterized by the activation of two types of glial cells: microglia and astrocytes [8]. Both microglia and astrocytes are involved in maintaining the homeostasis of the brain, but in the context of PD, their activation may contribute to inflammation and neuronal damage. Microglia are the resident immune cells of the brain, and their primary function is to surveil their surroundings for signs of injury, infection, or abnormalities. Oxidative stress triggers an immune response and activates microglia. Activated microglia release pro-inflammatory cytokines, chemokines, and ROS, contributing to neuroinflammation and further damage to the surrounding neurons. The prolonged activation of microglia can exacerbate the neurodegenerative process in PD. Another type of glial cell is astrocytes, which are the most abundant cells in the central nervous system and that provide support and nourishment to neurons. Astrocytes are involved in maintaining the blood–brain barrier (BBB), regulating neurotransmitter levels, and providing metabolic support to neurons. In PD, astrocytes also respond to the pathological changes such as oxidative stress and become hypertrophied reactive astrocytes. Eventually, reactive astrocytes form a gliotic scar, a dense network of astrocytes, in a process called astrogliosis, which can impede tissue repair and worsen neurodegeneration. On the other hand, reactive astrocytes are also involved in neuroprotection and the regeneration of the injured brain. Astrocytes rapidly respond to ROS and regulate microglial activation to protect neurons and suppress excessive inflammation. While glial activation is a common phenomenon in neurodegenerative disorders and the reactive astrocytes have been considered as a byproduct of neurodegeneration, the activation of both microglia and astrocytes may be the origin of neuronal dysfunction and degeneration in a non-cell autonomous manner [9,10].

Proinflammatory cytokines are signaling molecules that mediate communication between immune cells and contribute to the inflammatory response [11,12]. *Tumor necrosis factor-α* (TNF-α), *interleukin*-1β (IL-1β), and *interleukin-6* (IL-6) are among the key cytokines released during neuroinflammation in PD [13]. These mediators can induce oxidative stress, activate microglia and astrocytes, and contribute to the production of neurotoxic molecules, including nitric oxide and prostaglandins [14]. The sustained production of proinflammatory cytokines may also contribute to the production of ROS, which can damage neurons and exacerbate neurodegeneration and the progressive loss of dopaminergic neurons in the SN, a feature of PD.

Elevated levels of biogenic acetaldehyde and its derivatives are also strongly associated with various diseases, including PD, Alzheimer’s disease, and psychiatric disorders [15]. Oxidative stress is an imbalance between the production of ROS and the antioxidant capacity to detoxify the resulting damage. This oxidative damage can affect many intracellular molecules: proteins, nucleic acids, and lipids [16,17,18]. In particular, lipid peroxidation leads to the formation of highly reactive aldehyde species, such as malondialdehyde (MDA) and 4-hydroxy-2-nonenal (4-HNE) [19,20,21]. MDA is produced at high levels during lipid peroxidation and is used as a biomarker of oxidative stress. HNE, a bi-functional aldehyde, easily forms cross-links within or between proteins. This HNE–protein adduct would lead to a partial unfolding of proteins and the exposure of hydrophobic moieties on the protein surface resulting in initial protein aggregates [22]. The dopamine metabolite 3,4-dihydroxyphenylacetaldehyde (DOPAL) is known to be involved in catechol-induced neurotoxicity [23]. DOPAL is highly reactive and can covalently modify α-synuclein, thereby initiating proteostasis and the degeneration of dopaminergic neurons [24]. All reactive aldehydes are linked to aldehyde-induced protein modification and cellular damage, accelerating neuroinflammation and neurodegenerative processes. In addition, exposure to environmental toxins, i.e., rotenone and paraquat, generates reactive aldehydes and ROS, leading to PD-like symptoms in animal models [25]. Therefore, therapeutic approaches aimed at mitigating the toxic effects of aldehydes are a promising area in the pursuit of effective treatment of PD.

Aldehyde dehydrogenases (ALDHs) are involved in the detoxification of endogenous and exogenous aldehydes [26], including MDA, 4-HNE, and DOPAL [19,27,28]. Reduced ALDH activity has been implicated as a risk factor for the development of PD [29,30], leading to oxidative stress, protein misfolding, and neuronal damage, all of which are hallmarks of PD. Therefore, understanding the role of ALDH and the mechanisms involved in biogenic aldehyde production and detoxification may lead to the development of effective strategies for the prevention or treatment of these diseases.

Aldehyde-reducing composition (ARC) is an extract lysate of a novel yeast strain “Kwon P-1,2,3” that produces both ALDH and glutathione (GSH) with high efficiency [31]. This strain was generated by chemical mutagenesis rather than genetic recombination and was selected for GSH and ALDH in the presence of methylglyoxal and lysine. This mutant strain is generally recognized as safe (GRAS) in the use of food, health food, feeds, cosmetics, and medicines. Developed through optimized fermentation processes, ARC reduced acetaldehyde and malondialdehyde in alcohol-administered animal models [32], possibly containing a high ALDH activity with anti-aldehyde and antioxidant components. ARC is currently marketed as an alcohol hangover relief agent in the Republic of Korea, proving its safety in humans [32].

We previously reported that the inhibition of proinflammatory cytokines and normalization of microglia and astrocytes ultimately protected dopaminergic neurons from death in a mouse model of lipopolysaccharide (LPS)-induced neuroinflammation [33,34]. In this study, we found that ARC significantly reduced LPS-induced inflammation and oxidative stress in the brain and liver of mice. ARC inhibited LPS-induced microglial and astrocyte activation, proinflammatory cytokine release, and upregulated antioxidant genes. These results indicate that the inhibition of aldehyde formation with ARC may be useful in the prevention and treatment of neurodegenerative diseases.

## 2. Results

### 2.1. Antioxidant and Anti-Aldehyde Effects of ARC

**Figure 1:**
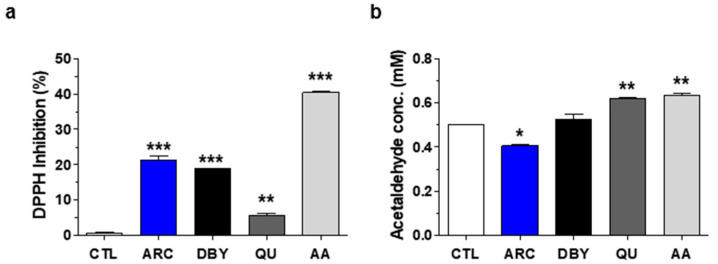
Antioxidant and anti-aldehyde effects of ARC. (**a**) Antioxidant effects. DPPH inhibition (%) of yeast extract ARC, inactive dry brewer’s yeast (DBY), quercetin (QU), and ascorbic acid (AA). (**b**) Anti-aldehyde effects. The concentration of 3,5-DHBA-acetaldehyde derivatives was determined in the presence of ARC, DBY, QU, or AA. Data are the mean ± SEM (*n =* 5). * *p* < 0.05, ** *p* < 0.01, *** *p* < 0.001 vs. CTL.

The antioxidant activity of ARC was compared with that of inactive dry brewer’s yeast (DBY), quercetin (QU), and ascorbic acid (AA) using the 2,2-diphenyl-1-picrylhydrazyl (DPPH) assay. ARC (2 mg/mL) significantly increased DPPH radical scavenging activity by 21.3%. The reference and positive antioxidant controls, 2 mg/mL DBY, 1 μg/mL QU, and 100 μM AA increased DPPH activity by 18.8%, 5.7%, and 40.4%, respectively (Figure 1a).

Similarly, the anti-aldehyde activity of ARC was compared with those of DBY, QU, and AA using the 3,5-dinitrobenzhydrazide (3,5-DHBA) assay. The 3,5-DHBA aldehyde quantification method is based on the spectrophotometric determination of hydrazones in an alkaline medium, producing the hydrazon-α-oxiazinic tautomer formed by the condensation of aldehydes and 3,5-DHBA [35]. ARC at 2 mg/mL significantly reduced the aldehyde concentration by 18.75%. However, the same concentrations of DBY, QU, and AA as above rather increased the aldehyde concentration by 5.06%, 23.72%, and 24.78%, respectively (Figure 1b).

### 2.2. Anti-Neuroinflammatory Effects of ARC in LPS-Induced Acute Neuroinflammation Mouse Model

#### 2.2.1. The Neuroprotective Effects of ARC on Dopaminergic Neurons

**Figure 2:**
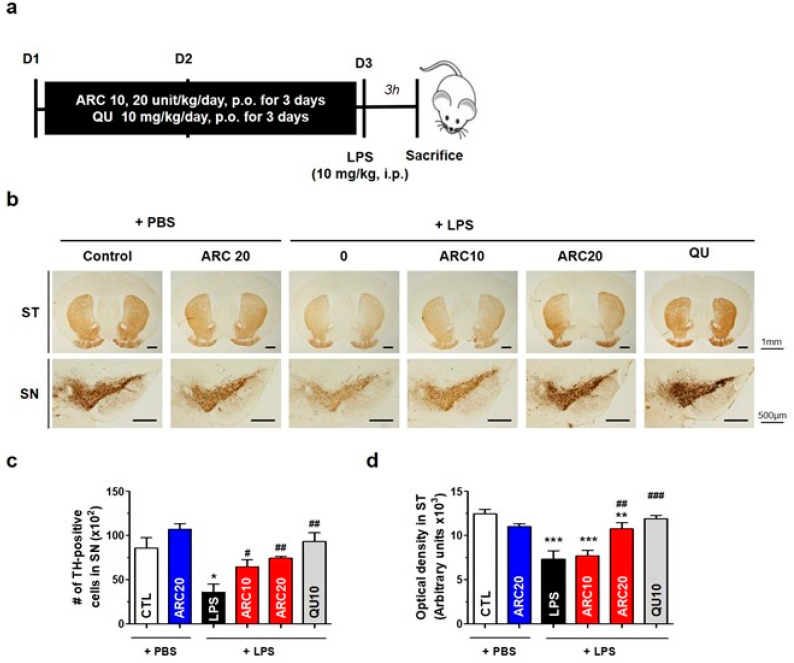
Protective effects of ARC on nigrostriatal dopaminergic neurons in LPS-injected mice. C57/BL6 mice (8 weeks, 19–23 g) were intraperitoneally injected with PBS (CTL), or LPS (10 mg/kg in PBS). ARC (0, 10, 20 units/kg/day) or QU (10 mg/kg/day, reference) was orally administered once a day for 3 days before the injection of LPS (10 mg/kg, *i.p*.) or PBS. At 3 h after LPS injection, mice were sacrificed and transcardially fixed. The brain sections were then prepared for immunohistochemical staining. (**a**) Experimental scheme. (**b**) Dopaminergic neurons were visualized by immunohistochemical staining with an anti-TH antibody in the ST and SN. (**c**) The number of TH-positive neurons and (**d**) optical density of TH-positive fibers in the ST was measured. Data represent the mean ± SEM (*n =* 5). * *p* < 0.05, ** *p* < 0.01, *** *p* < 0.001 vs. CTL, # *p* < 0.05, ## *p* < 0.01, ### *p* < 0.001 vs. LPS-injected vehicle.

Previously, we reported that the systemic injection of LPS at 10 mg/kg could activate astrocytes and microglia, resulting in the loss of tyrosine hydroxylase (TH)-positive neurons in the SN [33]. Based on these findings, we investigated the potential anti-neuroinflammatory effects of ARC in a mouse model of LPS-induced neuroinflammation. Mice were treated with ARC (0, 10, 20 units/kg/day) or QU (10 mg/kg/day) once daily for three days and then injected with LPS (10 mg/kg, *i.p.*) or PBS (Figure 2a for the experimental scheme). We then estimated the TH-positive neurons in the SN and ST regions of brain tissue sections. LPS-induced acute neuroinflammation decreased the total number of TH-positive cells in the SN and nerve fiber density in the ST (Figure 2).

The oral administration of ARC dose-dependently protected against the LPS-mediated loss of TH-positive dopaminergic neurons in the SN (*p* < 0.05 and *p* < 0.01, Figure 2b,c). The TH-positive optical density of nerve fibers in the ST was restored to near-normal levels by ARC at a dose of 20 units/kg (ARC20) (*p* < 0.01, Figure 2b,d). The efficacy of ARC at 20 units/kg was similar to that of the reference drug quercetin (10 mg/kg). Treatment with ARC (20 units/kg) alone without LPS injection did not alter the number of TH-positive cells in the SN and the intensity of TH-positive fibers in the ST, and no deleterious effect of ARC was observed.

#### 2.2.2. ARC Inhibits the Activation of Microglia and Astrocytes

The immunohistochemical staining of cryosections of brain tissue with anti-glial fibrillary acidic protein (GFAP, astrocytes) or anti-ionized calcium binding adaptor molecule 1 (Iba-1, microglia) antibodies allows astrocytes or microglia to be visualized. The activation of GFAP-positive and Iba-1-positive cells in LPS-injected brains is characterized by morphological changes such as enlarged cytoplasm, ramified processes, elongated dendrites, and thickened morphology. These activated microglia and astrocytes have been used as markers of neuroinflammation.

**Figure 3:**
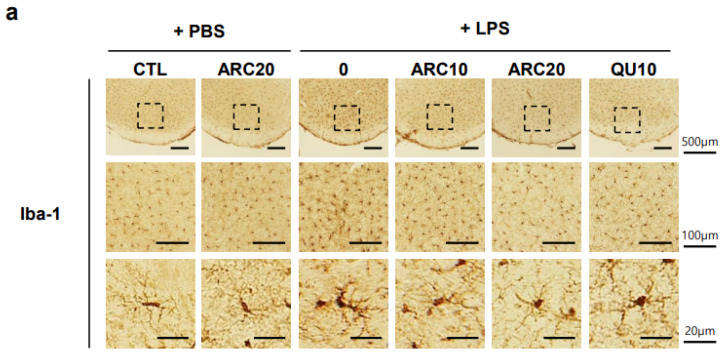

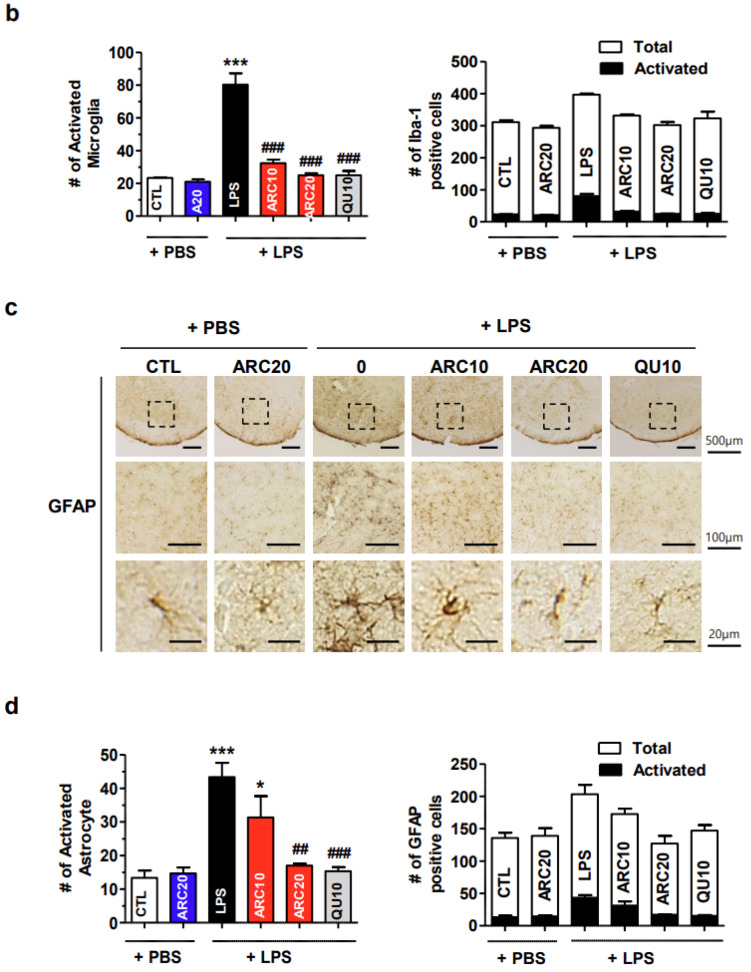
ARC suppresses LPS-induced activation of astrocytes and microglia in the SN. Mice brain sections were prepared as described in Figure 1. (**a**,**c**) Representative images of coronal brain sections containing the SN immunostained with anti-Iba-1 ((**a**), microglia) or anti-GFAP ((**c**), astrocytes) antibodies. Boxes in SN regions were enlarged to visualize activated microglia or astrocytes. (**b**,**d**) Quantitative analysis of microglia (**b**) or astrocytes (**d**). Upper graph, quantification of the number of activated microglia or astrocytes per section. Lower graph, comparison of the number of activated microglia or astrocytes (Activated) versus total Iba-1- or GFAP-positive cells (Total). Data represent the mean ± SEM (*n* = 5). * *p* < 0.05, *** *p* < 0.001 vs. CTL, ## *p* < 0.01, ### *p* < 0.001 vs. LPS-injected vehicle.

After LPS injection, the number of activated microglia and astrocytes in the SN region increased by 3.4- and 3.3-fold, respectively, compared to controls (Figure 3, *p* < 0.001), whereas the total number of microglia and astrocytes increased only 10% and 30%, respectively, compared to controls, and most Iba-1-positive microglia and GFAP-positive astrocytes exhibited a resting morphology with smaller cell bodies and fewer branching processes. The administration of ARC dose-dependently inhibited the LPS-induced activation of astrocytes and microglia in the SN (Figure 3, *p* < 0.05 vs. LPS-treated). The inhibitory effect of ARC at a dose of 20 units/kg (ARC20) on the activation of microglia and astrocytes was similar to that of the reference drug quercetin at a dose of 10 mg/kg (QU10, *p* < 0.001 vs. LPS-treated). Again, there was no significant effect on microglial and astrocyte activation in the group receiving ARC only (20 units/kg/day). Our results suggest that ARC has a potent anti-neuroinflammatory effect by inhibiting the activation of microglia and astrocytes in a dose-dependent manner.

### 2.3. Anti-Inflammatory and Antioxidant Effects of ARC in the Liver

#### 2.3.1. ARC Suppressed the mRNAs of LPS-Induced Inflammatory Cytokines

**Figure 4:**
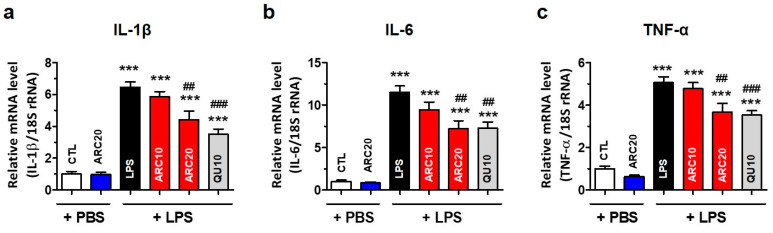
ARC suppresses pro-inflammatory cytokine mRNA expression in the liver of LPS-injected mice. Hepatic total RNAs of LPS-injected mice were isolated using Trizol reagent. Pro-inflammatory cytokine mRNA was quantified using real-time RT-qPCR. (**a**) IL-1β, (**b**) IL-6, and (**c**) TNF-α mRNA expression were graphed. Data represent the mean ± SEM (*n =* 5). *** *p* < 0.001 vs. CTL, ## *p* < 0.01, ### *p* < 0.001 vs. LPS-injected vehicle.

The intraperitoneal injection of LPS into mice induces a systemic inflammatory response and triggers an immune response, leading to the production of various cytokines [36]. The liver is the major organ damaged by LPS because it absorbs and eliminates injected LPS. Therefore, liver mRNA levels of inflammatory cytokines induced by LPS may be a good indicator of systemic inflammation. As a result of measuring liver mRNA levels by real-time RT-qPCR, IL-1β, IL-6, and TNF-α mRNA were found to increase by 6.5-fold, 11.5-fold, and 5.1-fold, respectively, in the LPS-injected group compared to the control group (*p* < 0.001, Figure 4). ARC treatment dose-dependently reduced, but did not completely normalize, the mRNA levels of these inflammatory cytokines (Figure 3). The reduction in IL-1β, IL-6, and TNF-α mRNA by ARC20 was similar to that of the reference drug quercetin (QU10). The administration of ARC20 alone, without LPS, did not alter the expression of inflammatory cytokines.

#### 2.3.2. Antioxidant Effects of ARC in the Liver

**Figure 5:**
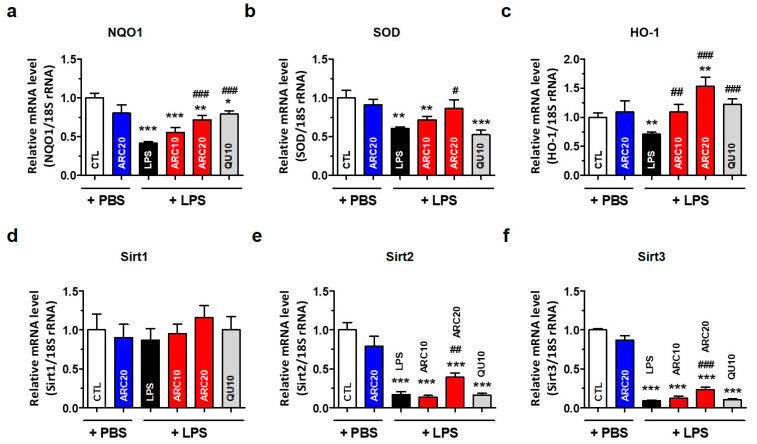
ARC increases the mRNA levels of antioxidant genes in the liver of LPS-injected mice. Hepatic total RNAs of LPS-injected mice were isolated using Trizol reagent. The mRNA levels of antioxidant genes were determined by real-time RT-qPCR. Relative mRNA levels of (**a**) NQO1, (**b**) SOD, (**c**) HO-1, (**d**) Sirt1, (**e**) Sirt2, and (**f**) Sirt3 were graphed. Data represent the mean ± SEM (*n =* 5). * *p* < 0.05, ** *p* < 0.01, *** *p* < 0.001 vs. CTL, # *p* < 0.05, ## *p* < 0.01, ### *p* < 0.001 vs. LPS-injected vehicle.

In addition to inducing an inflammatory response, LPS generates intracellular reactive oxygen species (ROS) and induces oxidative stress [17,37,38]. Increased oxidative stress leads to the decreased expression of antioxidative enzymes such as NAD(P)H quinone oxidoreductase 1 (NQO-1), superoxide dismutase (SOD), heme oxygenase (HO-1), and Sirt family genes [39]. To investigate the antioxidant effects of ARC, we measured the mRNA levels of NQO1, SOD, and HO-1 in the liver by real-time RT-qPCR. LPS decreased NQO1, SOD, and HO-1 mRNA by 59%, 39%, and 29%, respectively (Figure 5a–c). ARC treatment restored the mRNA expression of antioxidant genes to control their levels in a dose-dependent manner. It is noted that ARC20 increased HO-1 mRNA above the control level. Quercetin (10 mg/kg/day, QU10) normalized NQO-1 and HO-1 mRNA but not SOD.

We measured the hepatic mRNA levels of Sirt family genes and found that LPS caused a significant decrease in Sirt2 by 85% and Sirt3 by 90%, but not Sirt1 (Figure 5d,e). Only ARC20 slightly reversed the LPS-induced decrease in Sirt2 and Sirt3 mRNA, but QU10 had no effect on them. The changes in Sirt1 mRNA levels were not statistically significant for any treatments. ARC20 alone, without LPS, had no effect on the expression of antioxidant genes.

## 3. Discussion

This study demonstrated that a patented yeast extract lysate, ARC, which possesses potent antioxidant and anti-aldehyde activities, is a promising disease-modifying agent for the prevention and treatment of neuroinflammation-related diseases. In LPS-induced neuroinflammation in vivo, ARC effectively protected dopaminergic neurons from LPS-induced neuronal cell death, neuroinflammation, and inflammatory responses in the liver.

In this study, the antioxidant and anti-aldehyde activities of ARC yeast extract lysate were compared in vitro with three reference drugs: DBY, a normal yeast extract; ascorbic acid (AA), an antioxidant; and QU, an antioxidant and mitochondria-activating agent [33]. As shown in Figure 1, ARC exhibited 21% DPPH inhibition, half the efficacy of AA, while QU showed only 6% inhibition. On the other hand, anti-aldehyde activity was found only in ARC. Considering that QU, which has the least antioxidant activity, was excellent for protecting dopaminergic neurons and neuroinflammation, it appears that activities other than anti-oxidation may be important in the treatment of neuroinflammation. We speculated that the anti-aldehyde activity of ARC may be one of them, since ARC significantly prevented the elevation of serum aldehyde levels in a separate toxin-induced PD mouse model study (manuscript in preparation).

ALDH belongs to a gene superfamily of Phase 1 oxidizing enzyme responsible for the detoxification of biogenic and xenogenic aldehydes [29]. There are 19 ALDH genes in the human genome, but only ALDH2, located in the mitochondrial matrix, is known to be efficient in acetaldehyde metabolism because it has the 900-fold lower *Km* (~0.2 µM) for acetaldehyde compared to other ALDHs. In particular, ALDH2 plays a key role in the oxidation of 4-HNE, MDA, and DOPAL as well as environmental aldehydes from cigarette smoke and automobile exhaust. It is important to note that ~35–45% of East Asians (Korean, Chinese, Japanese, and Taiwanese) carry the ALDH2*2 polymorphic allele (rs671, E487K), which represents less than 50% of the wild-type ALDH activity in heterozygotes, and <1–4% of the wild-type in homozygotes.

A considerable amount of evidence has emerged suggesting a role for ALDH in human disease [29,30,40]. Brain ALDH converts DOPAL to 3,4-dihydroxyphenylacetic acid (DOPAC), which is rapidly removed from the cells. Reduced ALDH activity results in an accumulation of DOPAL, as well as oxidative stress, protein misfolding, and neuronal damage, all of which are hallmarks of PD. Indeed, ALDH knockout mice display increased formation of DOPAL and 4-HNE, neurodegeneration, and age-dependent motor dysfunction. The stereotaxic injection of DOPAL resulted in a selective loss of TH-positive neurons accompanied by gliosis in the SN [41], and DOPAL was shown to cause alpha-synuclein oligomerization in vitro and in cellular models [24]. Therefore, although there are not many genetic studies on the association of ALDH2*2 and PD/AD [42], it is reasonable to hypothesize that decreased ALDH activity may be important in PD development due to DOPAL accumulation [23]. The effects of ARC in models of LPS-induced neuroinflammation can be interpreted as occurring via aldehyde scavenging.

PD is clearly defined as a neurodegenerative disease with mitochondrial dysfunction and neuroinflammation. Neuronal mitochondrial dysfunction exacerbates oxidative stress and neuroinflammation, and conversely, infiltrated leukocytes and activated microglia damage neuronal mitochondria. Considering that ALDH activity is substantially decreased in the SN of PD patients [42] and that ALDH2 is localized in the mitochondrial matrix, mitochondrial dysfunction alone may reduce ALDH activity in the SN of PD brains, even in the absence of the ALDH2*2 allele.

A limitation of this study is that we cannot directly determine the concentration of DOPAL in the SN. Instead, we quantified TH-positive cells (Figure 2) and monitored the activation of Iba-1- and GFAP-positive cells based on morphological changes in vivo [33]. ARC pre-treatment efficiently decreased the LPS-induced activation of microglia (Iba-1 positive cells) and astrocytes (GFAP-positive cells) in the SN (Figure 3), where microglia initiate an inflammatory response by releasing proinflammatory cytokines and chemokines. Since AA has no anti-aldehyde activity, it was not tested in this study, although AA shows the highest antioxidant activity in vitro (Figure 1) and some effects on anti-PD or neurodegeneration [43]. Instead, QU at 10 mg/kg (QU10) was used as a reference because QU10 protects against TH-positive neuronal death and inhibits the activation of astrocytes and microglia in the brain of LPS-injected mice [33]. These anti-PD and anti-neuroinflammatory effects of QU can be explained by mitochondrial activation of QU, but not by aldehyde scavenging, since QU has no anti-aldehyde activity. Further studies are needed to determine whether the anti-neuroinflammatory effects of ARC are due to increased mitochondrial activity or a decreased DOPAL concentration in the SN.

Peripheral tissues, including the liver, have been studied as potential sources of biomarkers or insights into PD with the idea that changes in peripheral tissues may reflect systemic alterations associated with PD. We have reported that the mRNA levels of anti-oxidative enzymes and cytokines in the liver can reflect the systemic status of oxidative stress and inflammation in the mouse model [44]. In this study, we used the liver mRNA levels of inflammatory cytokines as an indirect indicator of systemic inflammation because serum inflammatory cytokine levels were changed similarly to hepatic cytokines [44]. However, the relationship between liver and brain tissues in terms of the mRNA expression of specific genes in PD is complex and not fully understood. Because systemic cytokines are known to damage the BBB and infiltrate the brain, cytokine mRNA levels using dissected brain tissue in in vivo models may not accurately represent cytokine levels in the brain. Glial cell activation via immunohistochemistry is an indirect way to determine the inflammatory response in the SN because different brain regions have different cytokine gene expression; therefore, the activation of microglia can be interpreted as an increase in inflammatory cytokines in the SN. Under various neurodegenerative disease conditions, activated microglia release inflammatory cytokines and neurotoxic substances such as IL-1β, IL-6, TNF-α, and nitric oxide (NO), resulting in progressive neuronal degeneration in PD [45,46]. We measured the mRNA levels of three cytokines (IL-1β, IL-6, TNF-α) and found ARC treatment significantly reduced the LPS-mediated increase in IL-1β, IL-6, and TNF-α mRNAs in the liver (Figure 4). This, together with our findings that ARC reduced the LPS-induced activation of microglia and astrocytes, suggests that ARC may also have reduced the cytokine levels in the SN.

For similar reasons, we measured the mRNA levels of three representative anti-oxidative enzymes (NQO-1, SOD, HO-1), and sirtuins (Sirt2, Sirt3) in the liver, but not the brain, of LPS-injected mice. In response to oxidative stress, cells use the antioxidant defense system to protect them from damage by scavenging ROS and maintaining redox homeostasis. Antioxidant defense enzymes such as NQO-1, SOD, and HO-1 have relatively long-half-lives and are not consumed during their antioxidant actions, inducing an adaptive response to oxidative stress through the Keap1/Nrf2/ARE signaling pathway. The dysregulation of antioxidant genes can lead to PD; thus, targeting the Keap1/Nrf2/ARE pathway is considered as a reasonable strategy for PD prevention and treatment [47]. ARC treatment significantly restored hepatic NQO-1, SOD, and HO-1 mRNAs to normal levels (Figure 5). From these results, we reasoned that a similar antioxidant action may occur in the brain. Meanwhile, sirtuins are known to influence aging, DNA repair, gene expression, metabolism, and stress responses [48], and Sirt2 and Sirt3 are also essential proteins that regulate cellular processes related to oxidative stress and redox balance. Sirt2 and Sirt3 have been investigated for their potential neuroprotective roles in PD [49,50]. LPS stimulation decreased Sirt2 and Sirt3 mRNA by 85–90%, and only ARC20 significantly restored these mRNA levels towards normal, whereas QU10 did not (Figure 5). Therefore, ARC activities other than antioxidant, such as restoring mitochondrial activity and anti-aldehyde activity, may be required to explain these results. Although changes in the mRNA expression of sirtuins in PD brains were not clearly reported, it is possible that the expression of these genes was also altered in the SN region as there are many reports of increased oxidative stress in PD brains.

Another limitation of the study is that the metabolic pathway of ARC and its implications for neuroprotection have yet been explored. Considering that the active components of ARC, GSH, and ALDH are likely undergo proteolysis, it is important to understand how these metabolites that cross the BBB contribute to the therapeutic effects. Extensive research is needed in the future.

Our results showed that ARC yeast extract with antioxidant and anti-aldehyde activities alleviated most pathological conditions in LPS-injected inflammatory models. ARC could be an excellent candidate for therapeutic efficacy by modifying the pathogenesis of neurodegenerative diseases, although further studies are needed to determine the active components of ARC.

## 4. Materials and Methods

### 4.1. Preparation of ARC Lysate

ARC is a proprietary, differentiated, patented strain based on the GRAS (generally recognized as safe) strain of Saccharomyces cerevisiae [31]. A standardized ARC was prepared by PICOENTECH Co., Ltd. (Seongnam-si, Republic of Korea) as described [32]. Briefly, ARC yeast was cultured for 24 h in an incubator using yeast extract peptone dextrose (YPD) medium containing 1% yeast extract, 2% glucose, and 1% peptone and then fermented for 48 h through a 5 L fermenter (Marado-05D-PS, CNS, Daejeon, Republic of Korea). A yeast pellet was obtained by centrifugation at 2000× *g* for 10 min in a high-speed centrifuge (Supra R22, Hanil Scientific Inc., Gimpo, Republic of Korea) and was frozen for 2 days in a cryogenic freezer (CLN-52U, Nihon freezer, Tokyo, Japan). The frozen yeast pellet was freeze-dried (FDU-7006, Operon, Gimpo, Republic of Korea) to obtain a yeast extract powder.

The yeast extract powder (3 g) was dissolved in 10 mL phosphate-buffered saline (PBS, pH 8.0) containing Pierce^TM^ protease inhibitor (A32955, Thermo Fisher Scientific, Lenexa, KS, USA). Glass beads of 0.5 mm (11079105, Biospec, OK, USA) were added and shredded with a bead homogenizer (Mixer Mill MM400, Retsch, Nordrhein-Westfalen, Germany). The crushed extract was centrifuged and the supernatant was separated, and the extract lysate was used for research.

Inactive dry brewer’s yeast (EKOPRODUKTAS, Panevėžys, Lithuania), ascorbic acid (Sigma, St. Louis, MO, USA), and quercetin (Wako, Osaka, Japan) were purchased from commercial sources.

### 4.2. ALDH Enzyme Activity Assay

The ARC lysate was subjected to centrifugation to separate only the supernatant, which was then transferred to a microtube (MCT-150-C, Axygen, CA, USA). After that, 10 µL sample was added to a 990 µL reaction solution prepared by mixing 50 mM of potassium phosphate buffer (pH 8.0), Nicotinamide adenine dinucleotide phosphate (NADP) (final conc. 3 mM) (10128058001, Roche, Basel, Switzerland), and acetaldehyde (final conc. 1.5 mM) (402788, Sigma-Aldrich, Burlington, MA, USA) and then reacted at 30 °C for 5 min. Afterwards, the change in absorbance was measured at 340 nm using a spectrophotometer (Mega-800, Scinco, Seoul, Republic of Korea). In total, 100 µL of ALDH-containing ARC yeast lysate and 1 mL of 1X Bradford reagent (Biosesang Inc., Yongin, Gyeonggi-do, Republic of Korea) were mixed, and mixture was stabilized at room temperature for 2 min. Thereafter, the total amount of protein was computed by measuring the absorbance change at 540 nm using a spectrophotometer. The amount of NADPH produced was corrected using the total amount of protein.

### 4.3. DPPH Free Radical Scavenging Activity Assay

The free radical scavenging activity of the sample was measured by the DPPH scavenging photometric assay [51]. Chemically, the odd electron of the nitrogen atom in 2,2-diphenyl-1-picrylhydrazyl (DPPH) is reduced by receiving a hydrogen atom from antioxidants to the corresponding hydrazine. When DPPH is added to the reaction mixture, the rate of reduction is used as an indicator of the antioxidant capacity. The reaction mixture was prepared by adding 100 µL of the sample to 900 µL of the DPPH solution (12.5 µg/mL (31.7 µM) DPPH in DMSO, 40 mM Tris-HCl, pH 7.4). Samples used were ARC lysate (2 mg/mL), and DBY (inactive dry brewer’s yeast, 2 mg/mL) and quercetin (1 µg/mL) and ascorbic acid (100 µM, 17.6 µg/mL) as the reference and the positive control. The reaction mixture was vortexed thoroughly and left in dark at room temperature for 30 min. Absorbance was measured with a UV-VIS spectrophotometer at 517 nm. DPPH inhibition (%) or DPPH radical scavenging activity (%) was calculated according to the formula below.

DPPH inhibition (%) = [1 − (S − S_0_)/(C − C_0_)] × 100

where C and C_0_ are absorbances of the DPPH and water without sample, and S and sample S_0_ are absorbances of the DPPH and water with sample, respectively.

### 4.4. Anti-Aldehyde Activity Assay

Anti-aldehyde activity was determined by the aldehyde quantification method using 3,5-dinitrobenzhydrazide (3,5-DHBA) [35]. This method is based on the spectrophotometric measurement of hydrazones in an alkaline medium, which yields hydrazon-α-oxiazinic tautomeric forms formed by the condensation of aldehydes and 3,5-DHBA. The reaction mixture was prepared by adding 40 µL of sample to 3960 µL of substrate solution (50 mM potassium phosphate buffer, pH 8.0, 2 mM NADP, 0.5 mM acetaldehyde). ARC lysate (2 mg/mL), DBY (inactive dry brewer’s yeast, 2 mg/mL), and quercetin (1 µg/mL) were used for the sample, and ascorbic acid (100 µM, 17.6 µg/mL) was used as a reference for anti-aldehyde activity. The mixture was reacted at 30 °C for 1 h. Then, 0.1 mL of 0.05 M Ethylenediaminetetraacetic acid (EDTA), 0.2 mL of 0.01 M 3,5-DHBA solution in dimethylformamide (DMF), 0.2 mL DMF, and 0.1 mL of 2.5 M sulfuric acid were added and the mixture was heated at 95 °C for 10 min in a water bath to induce the production of 3,5-DHBA-acetaldehyde derivatives. The solution was cooled to room temperature and 0.4 mL of 2.5 M NaOH solution was added. The absorbance was measured at 478 nm. The content of 3,5-DHBA-acetaldehyde derivatives was determined using a calibration curve with various concentrations of acetaldehyde under similar conditions.

### 4.5. Acute LPS-Injected Neuroinflammatory Mice Model

Eight-week-old C57/BL6 male mice weighing 19–23 g (Daehan Biolink Co., Ltd., Eumseong, Republic of Korea) were divided into 5 groups (*n =* 5/group). The groups included a PBS-injected vehicle control (CTL), a PBS-injected ARC (20 units/kg/day) control, and three LPS-injected groups pretreated with ARC (0, 10, 20 units/kg/day) or quercetin (reference drug, 10 mg/kg/day). Throughout the study, all animals had ad libitum access to food and water. The mice received oral administrations of ARC (0, 10, 20 units/kg/day) or quercetin (10 mg/kg/day) daily for 3 consecutive days. Subsequently, they were injected with LPS (10 mg/kg, i.p., Sigma, St. Louis, MO, USA) or PBS. Three hours after the injection of LPS or PBS, the mice were sacrificed. Post-fixed brain tissues were prepared for immunochemical staining. Liver and serum were isolated for RNA and MDA quantifications, immediately frozen under liquid nitrogen, and stored at −80 °C until further use. The maintenance and treatment of the animals adhered to the Principles of Laboratory Animal Care (NIH publication No. 85–23, revised 1985) and the Animal Care and Use Guidelines (KHSASP-20–163) of Kyung Hee University, Seoul, Republic of Korea.

### 4.6. Immunohistochemical Staining of Brain Sections

Brain tissues were processed according to previously outlined methods [33,34]. In brief, mice underwent transcardial perfusion, followed by fixation with 4% paraformaldehyde (PFA) dissolved in 0.1 M phosphate-buffered saline (PBS). Subsequently, dissected brains were post-fixed overnight in 4% PFA at 4 °C and preserved in a 30% sucrose solution. A cryostat (Microsystems AG, Leica, Wetzlar, Germany) was employed to obtain brain sections with a thickness of 30 μm.

For immunohistochemical staining, brain sections were incubated overnight at 4 °C with primary rabbit anti-tyrosine hydroxylase (TH, 1:2000; Pel-Freez Biologicals, Rogers, AR, USA) for dopaminergic neurons, rabbit anti-glial fibrillary acidic protein (anti-GFAP, 1:5000; Neuromics, Edina, MN, USA) or rabbit anti-ionized calcium binding adaptor molecule 1 (anti-Iba-1, 1:1000; Wako, Osaka, Japan), respectively. The sections were then subjected to biotinylated anti-rabbit IgG and avidin-biotin peroxidase complex (ABC) standard kit (Vector Laboratories, Burlingame, CA, USA) and visualized through peroxidase activity staining. To quantify GFAP- or Iba-1- or TH-positive cells, stained brain sections were captured using a bright-field microscope (Olympus Optical, Tokyo, Japan).

The total number of TH-immunopositive neurons was assessed as previously described [52]. TH-positive cells in the SN of both hemispheres (5 sections/series) were counted on comparable sections for all treatment subgroups throughout the entire nucleus. Counting frames (47.87 × 36.19 μm) were positioned at the intersections of a grid (frame size 150 × 150 µm) randomly placed over the section, with counting conducted using a ×100 objective. The estimate of the total number of neurons was calculated following the optical fractionator equation. TH-immunopositivity in the ST was measured by the optical density of TH-positive fibers at ×40 magnification using ImageJ software (version 1.53e, http://imageJ.nih.gov/ij, accessed on 5 December 2023, National Institutes of Health, Bethesda, MD, USA). Activated microglia and astrocytes were categorized and counted based on their morphologies as previously described [33,34].

### 4.7. Real-Time Reverse Transcriptase-Quantitative Polymerase Chain Reaction (RT-qPCR)

Quantitative real-time RT-qPCR was conducted using 1.5 μg of total RNA isolated using Trizol reagent (Invitrogen, Carlsbad, CA, USA) and 10 pM oligo dT primers (Invitrogen) as previously described [53]. The real-time RT-qPCR reactions were carried out with 2× AmpiGene^®^ qPCR green Mix Lo-ROX (Enzo Biochem, New York, NY, USA) on a Roter-Gene Q (Qiagen, Hilden, Germany). The mRNA quantification was normalized by concurrently measuring nuclear DNA encoding 18S rRNA. Relative gene expression was determined using the 2^−ΔΔ^Ct method and is presented as the fold change compared to that of the control condition [53]. Primer sequences for specific genes included IL-1β (5′-tac gag ccg tag ccc aaa ca-3′ and 5′-gat cgt aac gga agc gtg ga-3′ for murine), IL-6 (5′-ctg gag tac cat agc tac ctg g-3′ and 5′-gtc ctt agc cac tcc ttc tg-3′ for murine), TNFα (5′-gcc cag acc ctc aca ctc aga t-3′ and 5′-ttg tcc ctt gaa gag aac ctg-3′ for murine), and antioxidant genes Nrf2 (nuclear factor (erythroid-derived 2)-like 2) (5′-tgg agg cag cca tga ctg a-3′ and 5′-ctg ctt gtt ttc ggt att aag aca ct-3′ for murine), NQO-1 (5′-atg gga ggt ggt cga atc tga-3′ and 5′-gcc ttc ctt ata cgc cag aga tg-3′ for murine), SOD (5′-gcc cgc taa gtg ctg agt c-3′ and 5′-cca gaa gga taa cgg atg cca-3′ for murine), HO-1 (5′-aag ccg aga atg ctg agt tca-3′ and 5′-gcc gtg tag ata tgg tac aag ga-3′ for murine), Sirt1 (5′-gct gac gac ttc gac gac g-3′ and 5′-tcg gtc aac agg agg ttg tct-3′ for murine), Sirt2 (5′-gag ccg gac cga ttc aga c-3′ and 5′-aga cgc tcc ttt tgg gaa cc-3′ for murine), Sirt3 (5′-tac agg ccc aat gtc act ca-3′ and 5′-aca gac cgt gca tgt agc tg-3′ for murine), and 18S rRNA (5′-gag cga aag cat ttg cca ag-3′ and 5′-ggc atc gtt tat ggt cgg aa-3′ for both human and murine).

### 4.8. Statistical Analysis

Statistical data are expressed as the mean ± standard error of the mean (SEM). The collected data were subjected to a one-way analysis of variance (ANOVA) followed by Tukey’s test that was used to determine the significance between more than two groups. The analysis was carried using Graphpad InStat (GraphPad Software, Prism 8.0.1, San Diego, CA, USA). A *p* value < 0.05 was considered significant.

## 5. Conclusions

In conclusion, the research demonstrates that ARC has a neuroprotective effect in an LPS-induced neuropathic inflammation model by normalizing activated glial cells, possibly by suppressing the inflammatory response and oxidative stress. Therefore, it can be concluded that ARC is a promising candidate for disease-modifying therapy for neurodegenerative diseases including Parkinson’s disease.

## 6. Patents

As a result of the work reported in this manuscript, a Korean patent “Novel Aldehyde Dehydrogenase Composition for Improving Behavior and Motor Function.” (10-2020-0179587) was filed on 1 December 2022.

## Data Availability

Data are contained within the article.
